# Complete Genome and Plasmids Sequences of a Clinical *Proteus mirabilis* Isolate Producing Plasmid Mediated NDM-1 From Italy

**DOI:** 10.3390/microorganisms8030339

**Published:** 2020-02-28

**Authors:** Ibrahim Bitar, Vittoria Mattioni Marchetti, Alessandra Mercato, Elisabetta Nucleo, Adriano Anesi, Silvia Bracco, Vanina Rognoni, Jaroslav Hrabak, Roberta Migliavacca

**Affiliations:** 1 Department of Microbiology, Faculty of Medicine, University Hospital in Pilsen, Charles University, 32300 Pilsen, Czech Republic; Jaroslav.Hrabak@lfp.cuni.cz; 2 Biomedical Center, Faculty of Medicine in Pilsen, Charles University, 32300 Pilsen, Czech Republic; 3 Dipartimento di Scienze Clinico-Chirurgiche, Diagnostiche e Pediatriche, Università degli Studi di Pavia, 27100 Pavia, Italy; vittoria.mattionimarche01@universitadipavia.it (V.M.M.); alemercato93@gmail.com (A.M.); elisabetta.nucleo@unipv.it (E.N.); roberta.migliavacca@unipv.it (R.M.); 4 Laboratorio di Microbiologia, Azienda Socio Sanitaria Territoriale di Lodi, 26900 Lodi, Italy; adriano.anesi@asst-lodi.it (A.A.); silvia.bracco@asst-lodi.it (S.B.); Vanina.Rognoni@asst-lodi.it (V.R.)

**Keywords:** *Proteus mirabilis*, bla_NDM-1_, qnrD1

## Abstract

*Background:* The spread of carbapenemase genes, such as *bla*_NDM-1_, in *Proteus mirabilis* poses a public health threat. The aim of the study was to characterize the genome and plasmids sequences of an NDM-1-positive strain (IBCRE14), which was isolated in 2019 from a catheterized patient hospitalized in Italy. *Methods*: Whole genome sequencing (WGS) of IBCRE14 was performed on extracted genomic DNA using Sequel I platform. Genome assembly was performed using “Microbial Assembly”. Genomic analysis was conducted by uploading the contigs to ResFinder and PlasmidFinder databases from the Center for Genomic Epidemiology. *Results:* IBCRE14 had a genome size of 4,018,329 bp and harboured genes coding for resistance to aminoglycosides (*aadA1*), phenicol (*cat*), tetracycline (*tetJ*), and trimethoprim (*dfrA1*). A large plasmid (pIB_NDM_1) harboured antibiotic resistance genes against sulphonamide (*sul1*), trimethoprim (*dfrA14*), tetracycline (*tetB*), rifampicin (*arr-2*), aminoglycosides (*aadA1, aph3-VI*), and beta-lactams (*bla*_OXA-10_, *bla*_NDM-1_). Furthermore, a small plasmid (pIB_COL3M) harboured a *qnrD1* gene coding for quinolone resistance. *Conclusion:* The ability to conjugate and the presence of a composite antibiotic resistance island suggests that pIB_NDM_1 could both acquire more resistance genes and easily disseminate. To our knowledge, this is the first report on an untypable plasmid harbouring *bla*_NDM-1_ in *P. mirabilis,* in Italy.

## 1. Introduction

The Gram-negative rod *Proteus mirabilis* is a leading cause of urinary tract infections (UTIs), particularly in patients suffering from complicated or catheter associated UTIs [1]. Because of the pathogen’s intrinsic resistance to polymyxin, nitrofurantoin, and tigecycline [2], the additional acquisition of antimicrobial resistance genes represents an epidemiological and therapeutic threat [3]. Multi-drug resistance (MDR) profiles are constantly increasing worldwide in *P. mirabilis*, mainly due to the production of extended-spectrum β-lactamases (ESBLs), AmpC β-lactamases, and/or carbapenemases [4,5,6]. Since the 1990s, several metallo-β-lactamases (MβLs) encoded by mobile DNA have emerged in important Gram-negative pathogens (i.e., in *Enterobacterales*, *Pseudomonas aeruginosa*, and *Acinetobacter baumannii*) [7]. Few cases of *P. mirabilis* New Delhi metallo-beta-lactamase (NDM)-producer have been described in China [8], Brazil [9], Tunisia [10], Austria [11], India [12], and New Zealand [13]. In *Enterobacterales*, *bla*_NDM-1_ is one of the most spread MβL-encoding genes [2]. In *P. mirabilis*, the *bla*_NDM-1_ determinant has been frequently associated with plasmids of IncC, IncFII, IncR, or an unknown incompatibility group. The *bla*_NDM-1_ gene is mainly mobilized through the IS*Aba125*-bounded composite transposon Tn*125* [2]. The epidemiology and the impact of NDM-producing *P. mirabilis* strains on treatment outcomes is still widely unknown in Italy. In this study, we describe the first report of a *P. mirabilis* clinical strain carrying an untypable *bla*_NDM-1_ plasmid, in Italy.

## 2. Materials and Methods

### 2.1. Cases Presentation

On 11 February 2019, a 74-year-old male was hospitalized in the Internal Medicine Department of “Ospedale Maggiore” Hospital of Lodi (Northern Italy) due to a respiratory failure resulting from a chronic obstructive pulmonary disease (COPD). The anamnesis of the patient included dizzying syndrome, smoking, alcohol, chronic anaemia, high blood pressure, dyslipidaemia, and obesity. Antibiotic therapy at admission included intravenous (IV) azithromycin 500 mg pd for three days. On day 11, sepsis due to a urinary tract infection from *Enterococcus faecalis* was diagnosed, bladder catheter was removed, and the patient was treated by IV ampicillin 4 gr pd for 8 days. On day 18, intestinal colonization from vancomycin-resistant *Enterococcus faecium* (VRE) was recognised. The isolation of different *Enterococcus* species from the same patient can be explained by the administration of ampicillin-based therapy eventually selecting for the VRE isolates in the intestine detected through the rectal swab. On 2 March 2019, the patient was transferred to the Sub-Acute-Care Unit of the Long-Term Care Facility (LTCF) of “Sant’Angelo Lodigiano” Hospital (Lodi, Northern Italy) in order to continue medical care, and functional isolation was applied for VRE. On day 35, the patient developed a *Clostridioides difficile* diarrhoea, spatial isolation was applied, and oral vancomycin 2 gr pd was administered for 14 days. On 3 April 2019, the *P. mirabilis* IBCRE14 strain was isolated and recognized as *bla*NDM-type gene positive (Pm-NDM) by XpertCarba-R System (Cepheid, Sunnyvale, CA, USA) from urine and then from rectal swab. No treatment was administrated on the above catheterized patient. The patient was discharged on 17 June 2019.

### 2.2. Second Case

On 4 March 2019, a 93-year-old male was hospitalized in the Internal Medicine Department of “Ospedale Maggiore” Hospital of Lodi due to pneumonia. The anamnesis of the patient included high blood pressure, diabetes mellitus type 2, atrial fibrillation, prostatic hypertrophy, and cognitive decay. At the admission, antibiotic therapy was given (IV ceftriaxone 2 gr pd for 5 days plus IV azithromycin 500 mg pd for 3 days). Then, piperacillin-tazobactam was administered in place of ceftriaxone (13.5 gr pd for 7 days by IV infusion). An intestinal colonization from vancomycin-resistant *Enterococcus faecium* (VRE) was documented on day 14. On the same day, the patient was transferred to the Sant’Angelo LTCF Lodi, Northern Italy) to continue the medical treatment. Functional isolation of the patient was applied to avoid VRE dissemination. On day 33, a UTI was suspected and IV ceftriaxone 2 gr pd for 7 days was empirically administered. On 8 April 2019, a further *P. mirabilis*-NDM producing bacteria was identified from catheter urine and then from the surveillance rectal swab. Spatial isolation was applied, making cohort of the two colonized patients. The patient was discharged on 5 May 2019.

### 2.3. Bacterial Strains and Antimicrobial Susceptibility Testing

Thirty samples, in total, were then obtained by the hospital staff from room surfaces and patients’ skin using SRK Hygiene monitoring kit system (Copan Italia S.p.a, Bovezzo, Italy), in order to assess the extent of environmental contamination, and with containment purposes. A total of eight *P. mirabilis*-NDM producing strains collected from the urine (*n* = 2), rectal swabs (*n* = 2), and skin swabs (*n* = 2) of the two inpatients, as well as environmental swabs (door knobs) (*n* = 2), were stored after selection on CHROMagar™(Paris, France) CPE (BD, Becton Dickinson, New Jersey, USA) and identification, for further molecular characterization. *P. mirabilis* IBCRE14 strain was considered as representative for WGS characterization.

The *P. mirabilis* isolates, selected on CHROMagar™ CPE (BD), showed positivity to a *bla*NDM-type gene by XpertCarba-R System (Cepheid). Species identification was performed using matrix-assisted laser desorption ionization-time of flight mass spectrometry (MALDI-TOF MS) using MALDI Biotyper software (Brucker Daltonics, Bremen, Germany). Antimicrobial susceptibility test was assessed by Vitek-2 System (bioMerieux, Marcy-I’Etoile France) and confirmed by Microscan AutoScan-4 (Beckman-Coulter) and broth-microdilution method; results were interpreted according to EUCAST (http://www.eucast.org) [14].

### 2.4. Conjugation Assay

Conjugation experiment was performed by the liquid mating assay using *Escherichia coli* J63 (STR)r as a recipient in order to investigate whether the *bla*_NDM-1_ gene was located on a conjugative element. The transconjugants were selected on MacConkey agar (Scharlab, SL, Barcelona, Spain) plates supplemented with streptomycin (100 mg/L) (Sigma-Aldrich, St. Louis, Missouri, USA) and meropenem (Sigma-Aldrich) (2 mg/L). The presence of *bla*_NDM-1_ gene in both donor and transconjugant strains was confirmed with *bla*_NDM_ targeted PCR.

### 2.5. Pulsed Field Gel Electrophoresis (PFGE)

The *P. mirabilis* isolates were grown on MacConkey (Scharlab) agar at 37 °C for 18 h and then in Luria Bertani Broth (Liofilchem Diagnostic Ltd, Roseto degli Abruzzi TE, Italy,). The restriction digestion was performed using *SfiI*I (35 U/sample; Promega Corporation, Madison, Wisconsin, USA) at 37 °C for 20 h. Fragments were separated in a 1% (w/v) Pulsed Field Certified Agarose gel (Bio-Rad Laboratories, Inc. Hercules, California, USA) in a 0.5x Tris-Borate-EDTA (TBE )buffer on a CHEF MAPPER (Bio-Rad Laboratories, Inc. Hercules, California, USA) apparatus at 14 °C at 6 V/cm for 25 h with an initial pulse time of 9.06 s and a final pulse time of 1m34s. Lambda 48.5 kb ladder (New England BioLabs, Ipswich, Massachusetts, USA) were used as molecular size markers. The gels were stained with ethidium bromide (Sigma Aldrich,,St. Louis, Missouri USA ), digitally photographed with Gel Doc 2000 (Bio-Rad Laboratories, Inc.) and normalized as TIFF images. Dendrograms of strain relatedness were created with Fingerprinting II version 3.0 software (Bio-Rad Laboratories, Inc.) using UPGMA. The Dice correlation coefficient was used with a 1.2% position tolerance in order to analyse the similarities of the banding patterns; the strains were considered clonally related in the case of >85% similarity.

### 2.6. Whole Genome Sequencing (WGS)

Whole genomic DNA was extracted from IBCRE14 using NucleoSpin Microbial DNA kit (Macherey-Nagel, Duren, Germany) and sequenced on Sequel I machine (Pacific Biosciences, Menlo Park, California, USA) using long reads sequencing technology. Library preparation was done following the manufacturer’s recommendation for microbial multiplexing for the Express kit 2.0. The DNA was sheared using g-tubes (Covaris, Woburn, Massachusetts, USA), and no size selection was performed during the library preparations.

### 2.7. Genome and Plasmids Analysis

The “Microbial Assembly” pipeline offered by the software “SMRT Link v8.0” was used to perform the assembly of the genome with minimum seed coverage of 20×. The contigs were uploaded to ResFinder (https://cge.cbs.dtu.dk/services/ResFinder/) [15], PlasmidFinder (https://cge.cbs.dtu.dk/services/PlasmidFinder/) [16], and Phaster (https://phaster.ca) [17,18] for the detection of antibiotic resistance genes, plasmid replicons, and phages, respectively. Finally, the genome was annotated using the NCBI Prokaryotic Genome Annotation Pipeline (PGAP).

## 3. Results and Discussion

**Figure 1:**
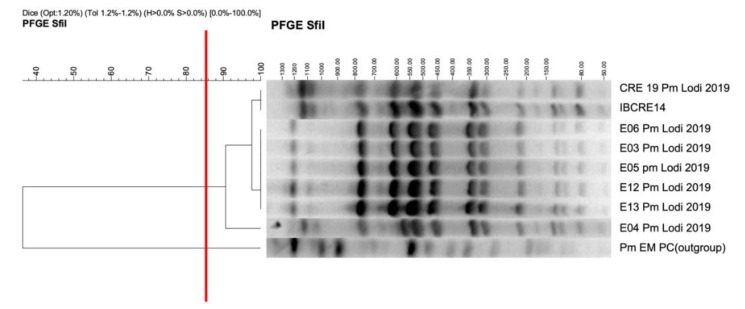
Dendrogram of seven out of the eight *P. mirabilis* strains along with the outgroup designated by Pm EM PC (*SfiI*; Dice coefficient: optimization 1.2% and tolerance 1.2%–1.2%). The cut-off to determine different clonal groups was established at 85%, which is designated by the vertical red line. The scale bar at the left represents similarity coefficient (%), while the one on the right represents fragments size (kb).

All eight of the *P. mirabilis* isolates showed the same antimicrobial-resistance profile observed for the IBCRE14 strain, showing resistance against ampicillin, piperacillin, third generation cephalosporins (cefotaxime, ceftazidime), nalidixic acid, meropenem, ertapenem, and tetracycline, but showing susceptibility to aztreonam, gentamicin, ciprofloxacin, and amikacin (Table 1). The (i) rapid *P. mirabilis*-NDM producing identification by phenotypic/molecular methods, (ii) patients cohorting, (iii) infection control measures undertaken (i.e., environmental sampling), and (iv) room disinfection by a dry mist of 12% hydrogen peroxide allowed for the resolution of the infection spread within a month. PCR and sequencing assays confirmed a *bla*_NDM-1_ gene variant. PFGE showed the presence of a unique profile (Figure 1) suggesting that infections were initiated by the same clone. Conjugation confirmed the transferability of *bla*_NDM-1_ and suggested the occurrence of a conjugative element (Table 1). The assembly resulted in three complete circular contigs: a 4018329 bp contig corresponding to the isolate genome, a 99278 bp contig corresponding to a plasmid harbouring the *bla*_NDM-1_, and a 2655 bp contig corresponding to a smaller plasmid.

IBCRE14 genome harboured genes coding for resistance to aminoglycosides (*aadA1*), phenicol (*cat*), tetracycline (*tet*), and trimethoprim (*dfrA1*). Moreover, the genome harboured four intact phages corresponding to a 30.3 Kb PHAGE_Salmon_Fels_2_NC_010463 phage, a 42.5 Kb PHAGE_Entero_mEp460_NC_019716 phage, a 68.6 Kb PHAGE_Yersin_PY54_NC_005069 phage, and a 68.6 Kb PHAGE_Salmon_vB_SosS_Oslo_NC_ 018279 phage coding for 39, 46, 54, and 90 phage proteins, respectively (integrase, terminase, portal, protease tail, head, capsid, virion, lysis, and plate). Upon blasting the regions corresponding to the phages against the NCBI database, all four of the phages were reported extensively in *P. mirabilis* genomes.

**Figure 2:**
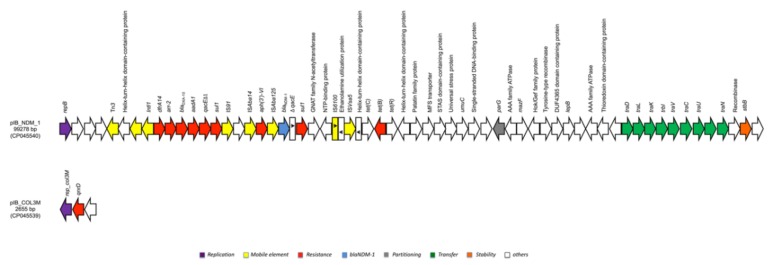
Linear map of pIB_NDM_1 and pIB_COL3M. Arrows show the direction of transcription of open reading frames (ORFs), while rectangles show truncated ORFs. Replicons, mobile elements, antibiotic resistance genes, *bla*_NDM_1_, partitioning genes, conjugal transfer genes, plasmid stability genes, and other remaining genes are designated by violet, yellow, red blue, grey, green, orange, and white, respectively.

The large plasmid (pIB_NDM_1) harboured antibiotic resistant genes against sulphonamide (*sul1*), trimethoprim (*dfrA14*), tetracycline (*tetB*), rifampicin (*arr-2*), aminoglycosides (*aadA1, aph(3′)-VI*), and beta-lactams (*bla*_OXA-10_, *bla*_NDM-1_). pIB_NDM_1 showed high scores when blasted against the NCBI database (https://blast.ncbi.nlm.nih.gov/Blast.cgi) with pPp47 (MG516912.1), a 142085 bp untypable plasmid detected in a *Proteus penneri* of animal origin isolated in Australia (sequence coverage of 91% and sequence identity of 99.99%) [19]. Plasmid analysis showed that the backbone harboured genes coding for replication (*repB*), conjugal transfer system (*tra* region), plasmid maintenance and stability (*parG*, *stb*), and type II toxin-antitoxin system (TA) (*mazF/mazE*). Moreover, the acquired region analysis showed a complex antibiotic resistance island (ARI): the ARI harboured three cassettes, suggesting the formation of a composite transposon. The first cassette carried an -*dfraA14* – *arr*-*2* – *bla*_OXA-10_ – *aadA1* – *qacEΔ1* – *sul1*- array flanked by a class 1 integron *Intl1* and an IS*91* transposase in opposite orientation. In proximity, the second cassette carried – *aph(3′)-VI* – IS*Aba125* -*bla*_NDM-1_ - *ΔqacE* – *sul1* –array flanked by the IS*Aba14* next to the *aph(3′)-VI* gene. The *bla*_NDM-1_ integration in this cassette is driven by IS*Aba125*, as reported elsewhere [3]. The last cassette, harbouring *tetB* gene and its regulators (*tetC* and *tetR*), was flanked by an IS*Vsa5* (Figure 2).

The small plasmid (pIB_COL3M) harboured a gene (*qnrD1*) coding for first generation quinolone resistance (and not second-generation quinolones such as fluoroquinolones). Only one plasmid replicon corresponding to pIB_COL3M was detected (COL3M), while pIB_NDM_1 did not belong to any of the known incompatibility groups present in the PlasmidFinder database. When blasted against the NCBI database, pIB_COL3M presented high similarity scores with a 2683 bp pCGP248 plasmid (JQ776503.1) described in a *P. mirabilis* strain of animal origin, in China [20]. The plasmid had three ORFs (open reading frames) corresponding to genes coding for replication (*rep_COLM3*), quinolone resistance (*qnrD1*), and for a hypothetical protein (Figure 2). Our results confirm the reports of the presence of *qnrD1* on a non-conjugative small plasmid that is found in high prevalence in *Proteeae* [20].

## 4. Conclusions

To our knowledge, this is the first report on a *P. mirabilis* clinical isolate carrying an untypable *bla*_NDM-1_ plasmid, in Italy. The presence of a complex ARI and conjugation ability confirm both the predisposition for further antibiotic-resistance genes acquisition and the potential for dissemination of the pIB_NDM_1 plasmid—a scenario that severely affects the therapeutic options against *Proteeae* infections. The findings highlight the importance of the screening for *P. mirabilis*-NDM -producers, an emerging threat in the Italian area. Moreover, the results underline the need for accurate infection control measures, as well as cohorting, in order to avoid the onset of outbreaks in clinical settings.

The genome sequence of IBCRE14 and the plasmid sequences of pIB_NDM_1 and pIB_COL3M have been deposited in GenBank under the accession numbers CP045538, CP045540, and CP045539, respectively.

## Figures and Tables

**Table 1:** Antimicrobial susceptibility profile of IBCRE14, the *Escherichia coli* J62, and the transconjugant J62/CRE14 that harbours the plasmid (pIB_NDM_1).

Isolate	MIC (mg/L)												
	AMP	SXT	CIP	GEN	TET	PIP	CTX	CAZ	MEM	ETP	TGC	TOB	NAL
IBCRE14	>128	>4	0.125	0.5	>32	64	>8	>16	16	2	1	0.5	64
J62	<1	0.031	<0.063	0.5	0.5	1	0.63	0.25	0.125	0.016	0.25	0.5	2
J62/CRE14	>128	>4	<0.063	0.5	>32	64	>8	>16	16	2	0.5	0.5	2

MIC, minimum inhibitory concentration; AMP, ampicillin; SXT, trimethoprim-sulfamethoxazole; CIP, ciprofloxacin; GEN, gentamicin; TET, tetracycline; PIP, piperacillin; CTX, cefotaxime; CAZ, ceftazidime; MEM, meropenem; ETP, ertapenem; TGC, tigecycline; TOB, tobramycin; NAL, nalidixic acid.
